# Targeting inflammation-metabolism crosstalk: current status and challenges of novel therapeutic strategies for osteoarthritis

**DOI:** 10.3389/fmed.2025.1754693

**Published:** 2026-01-12

**Authors:** Baolong Liu, Maoquan Zhang, Lu Gao, Hao Yang

**Affiliations:** 1 Qingdao Traditional Chinese Medicine Hospital, Qingdao Hiser Hospital Affiliated of Qingdao University, Qingdao, China; 2 Qingdao Women and Children’s Hospital, Qingdao, China

**Keywords:** inflammation, metabolism, novel strategies, osteoarthritis, targeted therapy

## Abstract

Osteoarthritis (OA) is a prevalent degenerative joint disease characterized by complex interactions between inflammation and metabolism. Recent years have seen a growing interest in targeted therapeutic strategies that address these interactions, aiming to alleviate the symptoms and progression of OA. This review provides a comprehensive overview of emerging treatment strategies in OA, focusing on the targeting of inflammatory mediators, metabolic regulators, and their combined applications. Additionally, we discuss the current challenges faced in the field, including the heterogeneity of OA and the need for personalized treatment approaches. By highlighting recent advancements and potential future directions, this article aims to contribute to the development of innovative therapies for OA, ultimately enhancing patient outcomes and quality of life.

## 1 Introduction

Osteoarthritis (OA), a prevalent degenerative joint disease and chronic condition characterized by complex interactions between inflammation and metabolism, as well as degeneration of articular cartilage, subchondral bone remodeling, and synovial inflammation, represents a major source of pain, disability, and socioeconomic cost worldwide due to its complex and multifactorial epidemiology involving genetic, biological, and biomechanical components, and leads to significant pain and functional impairment (1–3). The disease affects millions of people worldwide and is particularly prevalent among the elderly (4). Recent research has increasingly recognized the pivotal role of inflammation in OA pathogenesis, highlighting the intricate interplay between inflammatory processes and metabolic disturbances that drive disease progression. Inflammatory factors not only exacerbate joint damage but also disrupt metabolic homeostasis, creating a vicious cycle that fuels disease progression.

Inflammation was not initially regarded as a feature of OA (5). Following the identification of acute and chronic inflammation in osteoarthritis, this inflammation was initially considered a sequela rather than a disease driver–especially in contrast to prototypical inflammatory diseases such as rheumatoid or psoriatic arthritis–whereas recent preclinical and clinical evidence strongly supports inflammation as a central driver of osteoarthritis (6). OA inflammation is a complex pathophysiological phenomenon encompassing multiple cell and tissue types within and beyond the joint, and the interaction between inflammation and metabolism in OA is equally complex and multifaceted (7). Metabolic syndrome, characterized by obesity, insulin resistance, and dyslipidemia, has been linked to an increased risk of developing OA (8). Levels of inflammatory cytokines such as interleukin-6 (IL-6) and tumor necrosis factor-alpha (TNF-α) are elevated in OA conditions (9). Furthermore, metabolic dysregulation can enhance the inflammatory response, leading to further joint degradation. Recognition of these interactions has spurred the investigation of novel therapeutic approaches targeting both inflammatory and metabolic pathways to enhance outcomes in OA patients (10).

Recent advances in understanding the molecular mechanisms underlying OA have opened new therapeutic avenues. For instance, the role of the NLRP3 inflammasome in mediating metabolic inflammation has attracted significant attention given its function as a critical link between metabolic stress and inflammatory responses. Targeting this pathway may offer a strategy to alleviate both inflammation and associated metabolic dysfunctions contributing to OA. Additionally, dietary factors and lifestyle modifications, including exercise, have been shown to influence both inflammation and metabolic health, thereby further emphasizing the importance of a holistic approach to OA management (11).

Significant advancements have been made in unraveling the inflammation-metabolism interplay in osteoarthritis, including the identification of critical molecular mediators, regulatory signaling networks, and pathological crosstalk. However, translating these mechanistic insights into effective clinical interventions remains highly challenging. Key barriers include the complexity of multi-target interactions, disease heterogeneity across patient subsets, and discrepancies between preclinical model phenotypes and human OA pathophysiology. The heterogeneity of OA, shaped by genetic, environmental, and lifestyle factors, complicates the identification of universal therapeutic strategies (12). Furthermore, while some anti-inflammatory therapeutic agents have demonstrated efficacy, there remains a paucity of disease-modifying osteoarthritis drugs (DMOADs) that effectively halt disease progression (13). As research continues to evolve, it is imperative to focus on integrating anti-inflammatory and metabolic strategies to develop comprehensive treatment protocols for OA (14).

## 2 Main body

### 2.1 Pathophysiology of osteoarthritis

**FIGURE 1:**
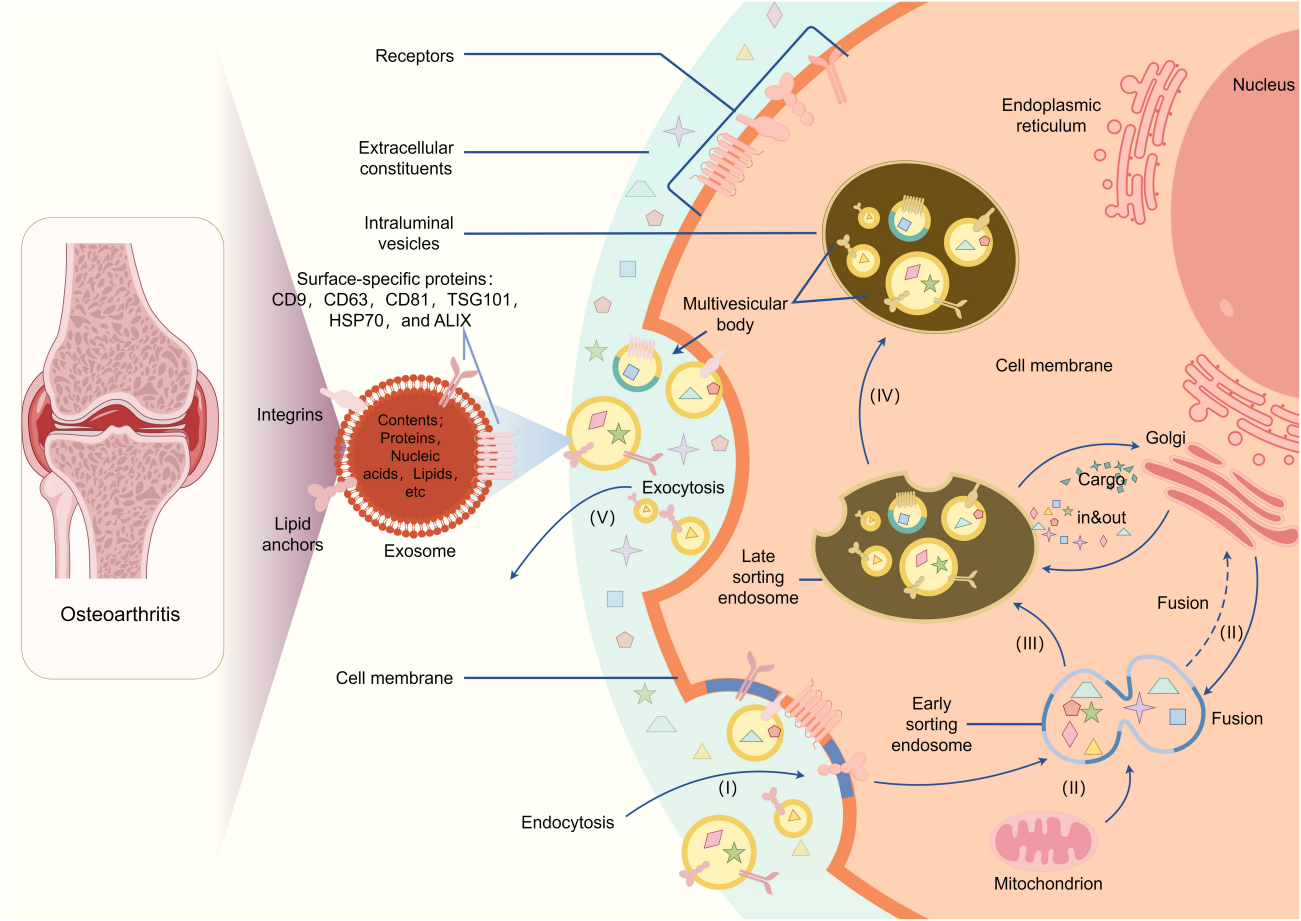
The pathophysiology of osteoarthritis.

Osteoarthritis (OA) is a highly prevalent degenerative joint disease that affects millions of individuals worldwide. It is characterized by progressive articular cartilage degradation, associated synovial inflammation, and aberrant subchondral bone remodeling. Collectively, these pathological changes drive joint pain, functional decline, and impaired quality of life (15). The pathophysiology of osteoarthritis (OA) is multifaceted and highly complex, encompassing a dynamic and intricate interplay of mechanical factors (e.g., abnormal joint loading, repetitive microtrauma), biochemical factors (e.g., imbalances in matrix-degrading enzymes and their inhibitors, dysregulated chondrocyte metabolism), and inflammatory factors (e.g., accumulation of proinflammatory cytokines, synovial immune cell infiltration) that collectively contribute to disease initiation and progression (Figure 1) (16). One of the primary features of OA is the breakdown of cartilage, which is essential for joint function. As the cartilage deteriorates, it leads to pain, stiffness, and loss of mobility, significantly impacting the quality of life for affected individuals. The disease is often associated with aging, obesity, and metabolic disorders, which further complicate its management and progression (17). Gaining a comprehensive understanding of the intricate underlying mechanisms driving osteoarthritis remains a pivotal prerequisite for the development of targeted and effective therapeutic strategies aimed at not only slowing or halting disease progression but also alleviating the debilitating symptoms, such as joint pain, stiffness, and functional impairment, that significantly impact patient quality of life (18, 19).

#### 2.1.1 Structure and function of articular cartilage

Articular cartilage, a specialized avascular connective tissue composed of chondrocytes and extracellular matrix (ECM; primarily type II collagen and proteoglycans), enables frictionless joint movement and shock absorption (20–22). Its avascular nature limits nutrient diffusion and repair capacity, rendering it susceptible to degeneration when exposed to stressors (such as inflammation, metabolic dysfunction) (23). OA is hallmarked by progressive ECM degradation [via matrix metalloproteinases (MMPs) and ADAMTS] and chondrocyte loss, leading to joint pain and functional decline (24).

#### 2.1.2 The role of inflammation in OA

Numerous key studies in recent years have demonstrated that inflammation plays a pivotal role in the pathogenesis of OA, challenging the traditional view that OA is a non-inflammatory disease. Low-grade chronic inflammation in OA is mediated by pro-inflammatory cytokines (IL-1β, TNF-α, IL-6), which activate chondrocyte catabolic pathways, induce MMP-1, -3, -13, and ADAMTS expression, and degrade type II collagen and aggrecan (25–27). Synovial inflammation (synovitis) further amplifies tissue damage by recruiting immune cells (macrophages) and releasing proteolytic enzymes, exacerbating pain and stiffness (28). This inflammatory cascade is not a secondary sequela but a primary driver of OA pathogenesis.

#### 2.1.3 The relationship between metabolic dysregulation and OA

**FIGURE 2:**
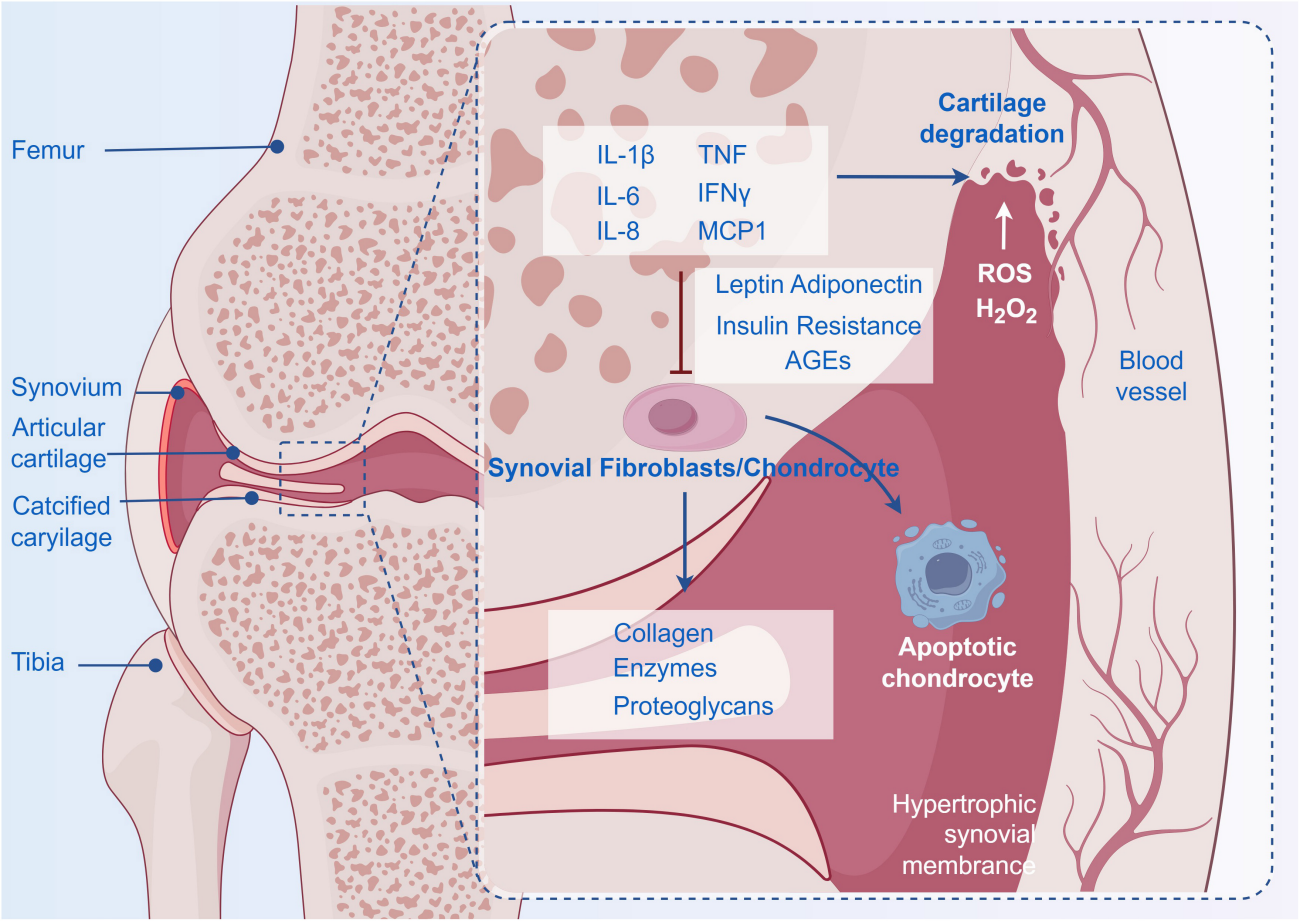
The relationship between metabolic dysregulation and OA.

Metabolic dysregulation–including conditions such as obesity, diabetes, and insulin resistance–has emerged as a critical driver of osteoarthritis (OA) pathogenesis. Notably, its role extends beyond mechanical stress, encompassing systemic inflammation and cellular dysfunction that contribute to disease progression (29–31). Obesity, a key metabolic risk factor, contributes to OA through adipokines such as leptin and adiponectin, which promote cartilage degradation and synovial inflammation (Figure 2) (32). For instance, leptin activates pro-inflammatory pathways in chondrocytes and synovial fibroblasts, while adiponectin’s protective role in cartilage homeostasis is compromised in obese individuals (33). Epidemiological studies highlight a threefold increased risk of knee OA in obese populations, underscoring the metabolic-mechanical interplay (34, 35). Diabetes mellitus, particularly type 2 diabetes (T2D), exacerbates OA through hyperglycemia-induced advanced glycation end products (AGEs) and insulin resistance (36, 37). AGEs disrupt cartilage matrix integrity by cross-linking collagen and activating receptor for AGE (RAGE)-mediated oxidative stress. Insulin resistance, a hallmark of T2D, impairs chondrocyte metabolism by downregulating anabolic pathways (such as type II collagen synthesis) and upregulating catabolic enzymes like matrix metalloproteinases (38). Mendelian randomization studies further suggest a causal link between insulin therapy and increased OA risk, possibly via augmented systemic inflammation (39). Metabolic inflammation, characterized by elevated IL-6, TNF-α, and complement components, bridges metabolic dysfunction and OA progression (40, 41). Adipose tissue-derived cytokines promote a pro-inflammatory microenvironment in joints, while systemic low-grade inflammation drives cartilage degradation and subchondral bone remodeling (42). Notably, synovial inflammation in OA is macrophage-predominant, releasing proteolytic enzymes that degrade the extracellular matrix (43–45). Mechanistically, metabolic dysregulation disrupts chondrocyte autophagy, a process critical for maintaining cartilage health. While autophagy initially protects against stress, chronic metabolic stress induces excessive autophagy in OA chondrocytes, leading to cell death and matrix loss (46). Adiponectin, through AMPK pathway activation, has been shown to restore autophagic balance in preclinical models, offering a potential therapeutic avenue. Emerging evidence also highlights the role of gut microbiome dysbiosis in metabolic OA, where microbial metabolites and leaky gut syndrome amplify systemic inflammation (47). Metabolomics studies further reveal distinct metabolic signatures in OA patients, including altered lipid and glucose profiles, which may serve as biomarkers for disease staging and personalized treatment. In conclusion, metabolic dysregulation profoundly influences OA pathogenesis through interconnected pathways of inflammation, adipokine imbalance, and cellular stress. Targeting these mechanisms–such as adipokine signaling, insulin sensitivity, and autophagic regulation–holds promise for developing disease-modifying therapies (48). Future research integrating multiomics approaches and longitudinal studies will deepen our understanding of this complex relationship, paving the way for precision medicine in OA management.

#### 2.1.4 OA heterogeneity: phenotype/endotype classification and therapeutic implications

Osteoarthritis is increasingly recognized as a highly heterogeneous disease with distinct phenotypic and endotypic characteristics, which directly influence disease progression patterns and therapeutic responses (49–52). Phenotypically, OA can be categorized based on clinical manifestations such as affected joint sites (knee, hip, hand), disease onset age (early-onset vs. late-onset), and presence of comorbidities (obesity, type 2 diabetes, metabolic syndrome) (8, 49, 53–58). However, phenotypic classification alone is insufficient to capture the underlying biological variability driving disease pathogenesis. Endotypic classification, which is rooted in distinct pathophysiological mechanisms, provides a more precise framework for guiding personalized therapy. Based on the relative contributions of inflammatory and metabolic dysregulation to disease initiation and progression, we propose a three-tier endotypic classification system for OA:

Inflammation-dominant OA: this endotype is characterized by robust synovial inflammation, elevated levels of pro-inflammatory cytokines (IL-1β, TNF-α, IL-6), and prominent synovitis observed via imaging or histology (50). It accounts for approximately 15%–30% of OA cases, often presenting with acute symptom flares, joint swelling, and rapid cartilage degradation (59, 60). Patients in this subgroup typically lack major metabolic comorbidities and exhibit strong responses to anti-inflammatory interventions in preclinical models.

Metabolism-dominant OA: this endotype is driven by systemic metabolic dysregulation, with obesity, insulin resistance, and dyslipidemia as core pathogenic factors (61–63). Key features include dysregulated adipokine signaling (leptin overexpression, adiponectin deficiency), advanced glycation end product (AGE) accumulation, and impaired chondrocyte metabolic homeostasis (64). Patients often present with gradual symptom progression, diffuse joint involvement, and a strong correlation between disease severity and metabolic parameters (body mass index, HbA1c, lipid profiles). Inflammatory markers are usually moderately elevated but not the primary driver of tissue damage (51).

Mixed OA: this endotype represents the most common subgroup (estimated 40%–60% of cases), characterized by intertwined inflammatory and metabolic dysregulation. In this subgroup, metabolic disturbances (e.g., insulin resistance) amplify inflammatory responses, while chronic inflammation further exacerbates metabolic dysfunction, forming a self-perpetuating vicious cycle. Patients typically present with both metabolic comorbidities and evidence of synovitis, with variable symptom patterns and disease progression rates.

The clinical implications of this endotypic classification are profound. For inflammation-dominant OA, targeted anti-inflammatory therapies such as IL-1β inhibitors (anakinra) or JAK inhibitors may be prioritized, as they directly address the core pathogenic driver. For metabolism-dominant OA, interventions targeting metabolic pathways–including insulin sensitizers (metformin), lipid-lowering agents (statins), and lifestyle modifications (weight loss, exercise)–are more likely to yield clinical benefits. The mixed endotype requires synergistic targeting of both pathways.

### 2.2 Targeted inflammatory factor treatment strategies

The treatment of inflammatory diseases has witnessed a marked shift toward precision-targeted strategies, which selectively target specific inflammatory cytokines, signaling molecules, or pathogenic pathways. This paradigm shift is driven by the recognition that conventional broad-spectrum anti-inflammatory therapies often exert off-target effects on homeostatic immune functions. Such off-target effects ultimately lead to compromised efficacy and increased adverse events in clinical practice. This targeted approach is particularly critical for advancing therapeutic development, as it enables the design of interventions that specifically disrupt pathological inflammatory cascades while sparing physiological immune responses, thereby minimizing systemic side effects and enhancing clinical efficacy. Central to this progress is the growing mechanistic understanding of inflammatory processes. This understanding encompasses the spatiotemporal dynamics of cytokine secretion, the interplay between pro-inflammatory and anti-inflammatory mediators, and the downstream signaling networks that amplify or resolve inflammation. Such mechanistic insight provides a rational basis for identifying actionable molecular targets in inflammatory disease research. Within this framework, this section will elaborate on three primary therapeutic strategies: cytokine-specific inhibitors (e.g., monoclonal antibodies against TNF-α or IL-6), which directly neutralize pro-inflammatory mediators; the clinical application of biologic agents (such as recombinant cytokines or cell-based therapies) that modulate immune cell function; and the development of small-molecule drugs designed to inhibit key enzymes or receptors within inflammatory signaling pathways (e.g., JAK inhibitors or MAPK antagonists).

#### 2.2.1 Cytokine inhibitors

Cytokine inhibitors have established themselves as a pivotal therapeutic modality in the management of diverse inflammatory disorders, and their utility in OA has been actively explored. These agents–encompassing monoclonal antibodies, fusion proteins, and small-molecule antagonists–exert their effects through selective targeting of key pro-inflammatory cytokines (TNF-α, IL-6, IL-1) that drive tissue inflammation, immune cell recruitment, and matrix degradation in OA pathogenesis. Preclinical evidence: TNF-α inhibitors (adalimumab) reduce cartilage degradation in mouse OA models, IL-1β inhibitors (anakinra) alleviate synovitis and chondrocyte catabolism. Clinical evidence: TNF inhibitors show inconsistent efficacy in Phase III OA trials due to heterogeneity, anakinra provides modest symptomatic relief in inflammation-dominant OA subsets (Phase II trials). IL-1β inhibitors such as anakinra have shown modest symptomatic relief in some OA subsets, particularly in patients with high synovial inflammation. The development of these inhibitors has been driven by the understanding of cytokine signaling pathways and the role of specific cytokines in OA pathology. However, challenges such as variable patient response, potential adverse effects (e.g., increased infection risk), and the need for individualized treatment strategies remain critical considerations in their clinical application.

#### 2.2.2 Application of biological agents

Biological agents, including monoclonal antibodies and fusion proteins, represent a significant advancement in the treatment of inflammatory diseases. These agents are designed to target specific components of the immune system, including cytokines, receptors, and immune cells, thereby modulating the inflammatory response. Preclinical evidence: Tocilizumab (IL-6 receptor antagonist) inhibits synovial inflammation and cartilage degradation in rat OA models. Clinical evidence: Tocilizumab is effective for rheumatoid arthritis and systemic juvenile idiopathic arthritis (Phase III trials), its OA efficacy is being evaluated in Phase II trials for inflammation-dominant subsets (65). Moreover, the use of biologics in inflammatory bowel disease has transformed treatment paradigms, allowing for targeted therapy that can induce and maintain remission in patients with moderate to severe disease (66). The precision of biological agents enables tailored treatment options that can significantly improve patient outcomes. However, the high cost of these therapies and their potential for immunogenicity pose challenges, necessitating ongoing research to optimize their clinical application.

#### 2.2.3 Development of small molecule drugs

The development of small-molecule drugs targeting inflammatory pathways has gained increasing momentum driven by their inherent potential for oral administration and lower production costs compared with biologics. These small-molecule drugs (targeting inflammatory pathways) can modulate multiple aspects of the inflammatory response, including the inhibition of key signaling pathways that are involved in cytokine production and immune cell activation (67). Preclinical evidence: JAK inhibitors reduce proinflammatory cytokine levels and cartilage degradation in mouse OA models. Clinical evidence: JAK inhibitors show efficacy for rheumatoid arthritis and atopic dermatitis (Phase III trials), OA trials (Phase II) demonstrate symptom improvement in inflammation-dominant patients (68). These small molecules have demonstrated efficacy in treating conditions such as rheumatoid arthritis and atopic dermatitis, providing an alternative for patients who may not respond to traditional therapies (69–71). Additionally, the inherent flexibility in the chemical design of these small-molecule agents (e.g., JAK inhibitors) enables the systematic optimization of key pharmacokinetic properties–including oral bioavailability, *in vivo* half-life, and target tissue penetration–thereby further enhancing their overall therapeutic potential (72). However, notable challenges still remain in ensuring high target specificity for the intended inflammatory signaling pathways and minimizing unintended off-target effects, as such off-target interactions may trigger a spectrum of adverse reactions that compromise treatment safety and patient tolerability (73). Against this backdrop of addressing existing challenges, ongoing research focuses on two core objectives: deciphering the precise mechanisms of action of small-molecule anti-inflammatory agents, and developing structurally novel small molecules with enhanced target specificity and reduced off-target risks. Such research continues to broaden and advance the therapeutic landscape for the effective management of inflammatory diseases.

### 2.3 Application of metabolic regulators in OA treatment

The application of metabolic regulators in the treatment of OA is gaining traction as research continues to unveil the complex interplay between metabolism and inflammation in joint diseases. Metabolic dysregulation, and specifically systemic metabolic dysregulation with a particular focus on impaired insulin sensitivity and dysregulated lipid metabolism, has been increasingly recognized to be intricately involved in both the pathogenesis initiation and progressive deterioration of OA (74–76). By strategically targeting the aforementioned metabolic pathways–those linked to impaired insulin sensitivity and dysregulated lipid metabolism–novel or optimized therapeutic strategies hold significant potential for managing OA. Specifically, these approaches may mitigate joint-localized inflammation associated with OA, enhance overall joint function, and attenuate the progressive deterioration of joint tissues in the disease (61, 75, 77). Recent preclinical and clinical studies have indicated that metabolic interventions targeting the aforementioned dysregulated metabolic pathways can modulate the pro-inflammatory milieu within OA-affected joints. This modulation presents novel therapeutic avenues for OA management. Notably, these avenues extend beyond the scope of conventional analgesics and anti-inflammatory agents (78–82).

#### 2.3.1 Insulin sensitivity and OA

Insulin sensitivity plays a crucial role in the occurrence, development, and progression of OA (39). Insulin resistance, which is often closely associated with obesity, metabolic syndrome, and other metabolic comorbidities in clinical practice, has been increasingly documented to be closely linked to increased systemic and local joint inflammation as well as the accelerated degradation of articular cartilage and subchondral bone (83). Studies indicate that elevated levels of insulin and glucose can exacerbate inflammatory responses in chondrocytes, leading to the production of pro-inflammatory cytokines and matrix-degrading enzymes that contribute to cartilage breakdown (84). Furthermore, metabolic disorders characterized by insulin resistance–commonly observed in individuals with long-term metabolic abnormalities–can lead to dysregulated lipid metabolism. This dysregulation involves the abnormal accumulation of free fatty acids and impaired lipid clearance in joint tissues. In turn, these changes exert a synergistic effect that further aggravates the pre-existing inflammatory state within the OA joint microenvironment (85). Improving insulin sensitivity can be achieved via targeted lifestyle interventions–including calorie-controlled, anti-inflammatory diets and regular moderate-intensity physical activity–and pharmacological agents such as metformin, which serves as a first-line therapeutic for type 2 diabetes mellitus in clinical practice (86). Such an approach may offer a dual clinical benefit: not only effectively managing dysregulated blood glucose levels in populations with insulin resistance, but also alleviating joint inflammation, synovial hyperplasia, and the consequent pain closely associated with osteoarthritis progression (86, 87). Preclinical and preliminary human studies have indicated metformin’s potential benefits for knee osteoarthritis (OA), including anti-inflammatory effects, cartilage preservation, and pain relief; thus, this community-based randomized double-blind placebo-controlled trial (*n* = 107) evaluated the 6-months efficacy of oral metformin (2000 mg/d) versus placebo in symptomatic knee OA patients with overweight or obesity (86). At the 6-months follow-up, the metformin group demonstrated a significantly greater reduction in knee pain as measured by the 100-mm visual analog scale (VAS) compared to the placebo group (mean between-group difference: −11.4 mm; 95% CI, −20.1 to −2.6; *P* = 0.01), with a standardized mean difference of 0.43 (95% CI, 0.02–0.83) and predominantly mild gastrointestinal adverse events. These findings support metformin as a therapeutic option for symptomatic knee OA in this patient population, though confirmation in larger-scale clinical trials is needed due to the modest sample size (86). This highlights the potential of targeting insulin sensitivity as a therapeutic strategy in OA management.

#### 2.3.2 Lipid metabolism and joint inflammation

Lipid metabolism is intricately linked with inflammatory processes in osteoarthritis (OA). Accumulating preclinical and clinical research consistently shows that dyslipidemia contributes actively to the sustained inflammatory response observed in OA. This condition is characterized by abnormal blood lipid profiles, including elevated low-density lipoprotein cholesterol (LDL-C), reduced high-density lipoprotein cholesterol (HDL-C), and increased triglycerides (88). Elevated levels of certain lipids, such as triglycerides and low-density lipoprotein cholesterol (LDL-C), have been associated with increased inflammation and pain in OA patients (89). Moreover, specific fatty acids, including omega-3 and omega-6 fatty acids, can modulate inflammatory pathways, suggesting that dietary interventions aimed at restoring healthy lipid profiles may be beneficial in managing OA symptoms (90). The role of adipokines, which are secreted by adipose tissue and influence both metabolism and inflammation, further complicates this relationship. In conclusion, targeting lipid metabolism in osteoarthritis (OA) could involve specific dietary modifications and clinically available pharmacological agents. Dietary strategies include low-saturated fat diets rich in omega-3 polyunsaturated fatty acids, while pharmacological options encompass lipid-lowering drugs such as statins or PPARγ agonists. This multifaceted therapeutic approach addresses lipid dysregulation in OA pathogenesis, thereby reducing synovial and systemic inflammation and improving joint structural integrity and functional status in affected patients.

#### 2.3.3 Targeted intervention in metabolic pathways

Targeting key metabolic pathways for intervention in OA treatment is an emerging and clinically relevant therapeutic strategy. These pathways include insulin signaling, lipid metabolism, and energy homeostasis. This approach holds considerable promise for effectively altering the progressive, degenerative course of OA. Recent advancements in understanding the metabolic reprogramming that occurs in OA have identified several key pathways, including glycolysis and fatty acid oxidation, that can be manipulated for therapeutic benefit (78). For instance, enhancing mitochondrial function and promoting fatty acid oxidation has been shown to alleviate inflammation and improve chondrocyte function (91, 92). Additionally, clinically investigated pharmacological agents target specific metabolic pathways in OA, including energy metabolism and lipid homeostasis. Examples of such agents include AMPK activators, such as metformin or resveratrol. These compounds have demonstrated considerable therapeutic potential: they reduce synovial and chondrocyte inflammation while promoting metabolic balance within OA joint microenvironments (93). Furthermore, the integration of lifestyle modifications, such as weight management and exercise, can synergistically enhance the effects of metabolic interventions, leading to improved outcomes for OA patients (94). This multifaceted approach underscores the importance of a comprehensive understanding of metabolic pathways in developing effective treatment strategies for OA.

### 2.4 Research progress on combination therapy strategies

Combination therapy strategies integrating interventions with distinct mechanisms of action have gained considerable traction in recent preclinical research and clinical practice. This is particularly relevant for managing multifactorial complex diseases, such as osteoarthritis or metabolic syndrome-related inflammatory disorders. These conditions are driven by core pathological mechanisms–intertwined inflammatory cascades and dysregulated metabolic pathways (62, 95). The integration of multiple therapeutic approaches aims to enhance efficacy and minimize adverse effects, providing a more comprehensive treatment framework. This section will specifically delve into recent advancements in metabolic-inflammation targeted combination therapy, focusing on the complex interplay between dysregulated inflammatory cascades and metabolic reprogramming, the efficacy and safety outcomes of phase II/III clinical trials, and promising future research directions centered on personalized therapeutic strategies.

#### 2.4.1 Synergistic targeting of inflammation and metabolism

Recent preclinical and clinical studies have highlighted the intricate bidirectional relationship between sustained inflammatory responses and metabolic dysregulation–including insulin resistance, dyslipidemia, and impaired glucose metabolism–particularly in prevalent metabolic-related conditions such as obesity and type 2 diabetes mellitus (T2DM) (95). Metabolic inflammation, often characterized by chronic low-grade inflammation, plays a crucial role in the pathogenesis of these diseases (96). For instance, adipose tissue releases various adipokines that not only regulate metabolism but also orchestrate inflammatory responses, thereby creating a feedback loop that exacerbates metabolic disorders (97). Targeting both key inflammatory pathways (e.g., NF-κB, NLRP3 inflammasome) and dysregulated metabolic processes (e.g., impaired glucose homeostasis, abnormal lipid metabolism) has emerged as a promising therapeutic strategy to mitigate the adverse clinical effects of metabolic-related diseases such as obesity, T2DM, and cardiovascular diseases (98–100). For example, therapies that inhibit pro-inflammatory cytokines such as IL-1β have shown potential in reducing insulin resistance and improving overall metabolic health (101). Furthermore, the gut microbiota has been identified as a critical player in mediating metabolic inflammation, suggesting that interventions aimed at modulating gut health may also yield beneficial effects on metabolic and inflammatory outcomes (102). The synergistic targeting of inflammatory and metabolic pathways not only ameliorates clinical symptoms but also directly targets the root pathological mechanisms driving disease progression, thereby paving the way for more mechanistically informed and clinically effective therapeutic strategies in metabolic-related disorders (98, 103, 104).

#### 2.4.2 Combination therapies in clinical trials

The landscape of clinical trials for combination therapies has expanded significantly, reflecting the growing recognition of their potential benefits. Recent phase II/III clinical trials have explored various combinations of existing therapies, including anti-inflammatory agents [e.g., non-steroidal anti-inflammatory drugs (NSAIDs) or interleukin-1 (IL-1) inhibitors] and metabolic modulators [e.g., glucagon-like peptide-1 (GLP-1) receptor agonists or AMP-activated protein kinase (AMPK) activators], to enhance overall treatment efficacy–particularly in improving clinical outcomes and delaying disease progression–in metabolic-related inflammatory disorders (86, 105–107). For instance, studies have demonstrated that the combination of anti-inflammatory drugs with lifestyle interventions, such as dietary modifications and exercise, can lead to improved metabolic outcomes in patients with obesity and type 2 diabetes (108). Additionally, the integration of novel agents targeting specific inflammatory pathways alongside traditional metabolic therapies is being actively investigated. Phase II/III trials are crucial for establishing robust evidence regarding the long-term safety and clinical efficacy of these multimodal combination approaches. This is because such regimens frequently involve complex interactions between the integrated therapeutic agents, including pharmacokinetic (PK) interactions and pharmacodynamic (PD) crosstalk. Specifically, PK interactions may include altered drug absorption or clearance, while PD crosstalk can manifest as synergistic or antagonistic effects on target pathways. The results from these trials are expected to provide valuable insights into optimal dosing regimens, treatment sequences, and patient selection criteria, ultimately guiding clinical practice toward more personalized medicine strategies.

#### 2.4.3 Future research directions

Moving forward, future research in combination therapy strategies for metabolic-related inflammatory disorders should focus on several mechanism-oriented key areas to further maximize their potential benefits in optimizing clinical efficacy and retarding disease progression. First, there is a pressing need for more robust preclinical models that accurately reflect the complexities of human diseases, particularly those involving metabolic and inflammatory interactions. These translationally relevant preclinical models can effectively facilitate the identification of mechanistically relevant novel therapeutic targets and the systematic evaluation of rationally designed combination strategies prior to their advancement into phase I/II clinical trials (109). Second, the integration of advanced technologies, such as machine learning and biomarker discovery, can enhance the precision of patient stratification and treatment monitoring, allowing for more tailored therapeutic approaches (108). Furthermore, exploring the role of lifestyle factors, such as diet and physical activity, in conjunction with pharmacological interventions will be essential in developing holistic treatment strategies that address both metabolic and inflammatory components of disease. Finally, collaborative efforts among researchers, clinicians, and industry stakeholders will be vital in translating findings from the laboratory to clinical practice, ensuring that combination therapies are effectively integrated into standard care protocols for patients with complex diseases.

### 2.5 Anti-inflammatory drugs in clinical trials for OA

Despite the well-established role of inflammation in OA pathogenesis and promising preclinical results, numerous anti-inflammatory agents–including cytokine inhibitors, biologics, and small-molecule anti-inflammatory drugs–have failed to demonstrate consistent efficacy in phase II/III clinical trials or failed to gain approval as DMOADs (110, 111). This section systematically analyzes the core factors contributing to these trial failures, providing critical insights for future therapeutic development.

#### 2.5.1 Inadequate targeting of OA heterogeneity

Osteoarthritis is a highly heterogeneous disease characterized by diverse endotypes driven by distinct pathophysiological mechanisms. As proposed earlier, OA can be classified into inflammation-dominant, metabolism-dominant, and mixed endotypes based on the relative contributions of inflammatory and metabolic dysregulation. However, most previous anti-inflammatory trials adopted a “one-size-fits-all” approach, enrolling heterogeneous patient populations without stratification by inflammatory endotype. For example, TNF-α inhibitors (e.g., adalimumab) that show robust efficacy in RA have failed to demonstrate significant structural benefits in OA trials (112). This failure is partly attributed to the fact that only a subset of OA patients (estimated 15%–30%) exhibit high levels of synovial inflammation and TNF-α expression (113). In contrast, patients with metabolism-dominant OA (e.g., obesity-related OA) may not respond to anti-TNF therapy, as their disease progression is primarily driven by metabolic dysregulation and adipokine imbalance rather than classical pro-inflammatory cytokines (114, 115). The lack of validated biomarkers to identify inflammation-dominant OA patients has led to the inclusion of non-responsive individuals in trials, diluting therapeutic effects and leading to negative outcomes.

#### 2.5.2 Overlooking the bidirectional crosstalk between inflammation and metabolism

Previous anti-inflammatory strategies focused solely on neutralizing pro-inflammatory mediators without addressing their interdependence with metabolic pathways. As highlighted in Section “2.1.3 The relationship between metabolic dysregulation and OA,” inflammation and metabolism form a vicious cycle in OA: inflammatory cytokines (such as IL-1β, TNF-α) disrupt insulin sensitivity and lipid metabolism, while metabolic dysregulation (e.g., insulin resistance, dyslipidemia) amplifies inflammatory responses. For instance, IL-1β inhibitors can temporarily reduce synovial inflammation but fail to reverse insulin resistance-induced chondrocyte catabolism or lipid accumulation in joint tissues (116). Conversely, metabolic stress (such as hyperglycemia, free fatty acid accumulation) can reactivate inflammatory pathways even after cytokine neutralization. This unidirectional targeting of inflammation, without concurrent modulation of metabolic pathways, limits long-term therapeutic efficacy and fails to halt disease progression. A striking example is the failure of IL-6 inhibitors in OA trials: while IL-6 is elevated in OA, its pro-inflammatory effects are tightly linked to insulin resistance and adipokine dysregulation (117, 118). Without addressing these metabolic co-factors, anti-IL-6 therapy cannot break the inflammation-metabolism cycle, leading to transient symptom relief but no structural protection.

#### 2.5.3 Mismatches between preclinical models and human OA phenotypes

Most preclinical OA models [such as destabilization of the medial meniscus (DMM) in mice, collagenase-induced OA] rely on acute mechanical injury or direct inflammatory stimulation, which poorly recapitulate the chronic, low-grade inflammation and metabolic dysregulation observed in human OA. These models often exhibit robust and synchronized inflammatory responses that are highly responsive to anti-inflammatory agents, creating a false sense of efficacy. For example, in the DMM model, IL-1β inhibition significantly reduces cartilage degradation, but this effect does not translate to human OA, where inflammation is milder, chronic, and intertwined with metabolic disturbances. Additionally, preclinical models rarely incorporate metabolic comorbidities (e.g., obesity, T2D) that are prevalent in human OA patients. As a result, drugs effective in lean, injury-induced OA models may fail in clinical trials involving obese or diabetic OA patients, who exhibit distinct inflammatory-metabolic crosstalk. The lack of translationally relevant preclinical models that recapitulate human OA heterogeneity and metabolic comorbidities has led to the advancement of drugs with poor clinical predictability.

#### 2.5.4 Insufficient focus on structural endpoints and disease staging

Many early anti-inflammatory trials primarily evaluated symptomatic outcomes (such as pain, function) rather than structural endpoints (e.g., cartilage thickness, joint space narrowing), which are critical for DMOAD approval. While some agents (such as NSAIDs, short-term IL-1 receptor antagonists) provide temporary pain relief, they fail to slow or halt cartilage degradation. Moreover, OA trials often enroll patients with advanced disease (Kellgren-Lawrence grade 3-4), where significant cartilage loss and subchondral bone remodeling have already occurred. Anti-inflammatory agents may be ineffective in reversing established structural damage, as their optimal window of action is likely in early-stage OA (grade 1-2) when inflammation-metabolism crosstalk is initiating tissue damage. The enrollment of late-stage patients in trials has further contributed to negative structural outcomes.

## 3 Discussion

The in-depth exploration of targeted therapeutic strategies that specifically address the interplay between inflammation and metabolism in the pathogenesis of OA offers promising new avenues for the clinical management of this degenerative joint disease (119–122). As our understanding of the complex pathophysiological mechanisms underlying OA continues to evolve and deepen, it has become increasingly evident in both preclinical studies and clinical observations that the intricate intersection of inflammatory signaling cascades and intracellular metabolic pathways plays a pivotal and multifaceted role in driving OA disease progression, exacerbating joint tissue degradation, and modulating patient symptomatology (123–125). Despite the notable advancements that have been achieved in basic and preclinical research on various diseases, significant challenges still remain in translating these promising mechanistic and experimental findings into effective, evidence-based clinical applications that can ultimately improve patient outcomes (124, 126, 127). For advancing understanding in this complex field, balancing diverse research perspectives and findings is of critical importance. Current literature emphasizes that multiple inflammatory mediators and metabolic signals interact through mechanisms that remain incompletely characterized. This underscores the need for a multidisciplinary approach, one that integrates insights from immunology, metabolism, and clinical practice. Future studies should prioritize elucidating these intricate interactions, as this may facilitate the identification of novel biomarkers and therapeutic targets. Moreover, the heterogeneity of OA represents a key challenge in optimizing clinical outcomes, as it manifests across multiple interrelated dimensions: patient demographics (encompassing age, sex, ethnic background, and lifestyle factors such as body mass index and physical activity levels), disease stages (ranging from early cartilage matrix degradation with minimal joint space narrowing to advanced synovitis, subchondral bone sclerosis, and joint deformity), and comorbidities (including type 2 diabetes mellitus, cardiovascular disease, and metabolic syndrome–conditions that may exacerbate OA pathogenesis by modulating systemic and local inflammatory responses). This multidimensional heterogeneity directly undermines the efficacy of conventional “one-size-fits-all” treatment approaches, as patients with seemingly similar OA phenotypes often exhibit divergent responses to pharmacologic (e.g., non-steroidal anti-inflammatory drugs) and non-pharmacologic (e.g., physical therapy) interventions (128–130). The failure of numerous anti-inflammatory agents in OA trials, as analyzed in Section “2.5 Anti-inflammatory drugs in clinical trials for OA,” further highlights the limitations of unidirectional, non-stratified therapeutic strategies. To overcome these challenges, future trials must adopt a precision medicine approach: first, validate multi-omics biomarkers (genomic, metabolomic, inflammatory) to stratify patients by endotype, second, design combination therapies that synergistically target both inflammatory and metabolic pathways to break their vicious cycle, and third, develop preclinical models that recapitulate human OA heterogeneity and metabolic comorbidities. For example, combining JAK inhibitors (anti-inflammatory) with metformin (insulin sensitizer) has shown synergistic benefits in preclinical models of obesity-related OA, and such combinations merit clinical investigation in metabolism-inflammation mixed endotype patients. Against this backdrop, there is an urgent need to advance personalized treatment strategies for OA. Future research should prioritize the stratification of OA patient populations based on integrated profiles of specific inflammatory mediators (e.g., interleukin-6, tumor necrosis factor-α, and matrix metalloproteinases) and metabolic markers (e.g., serum adiponectin, insulin resistance indices, and lipid metabolites). Such stratification would not only enable the identification of distinct OA endotypes–each driven by unique pathophysiological mechanisms (e.g., inflammation-dominant vs. metabolism-dominant)–but also facilitate the development of tailored interventions. For instance, patients characterized by a proinflammatory endotype might benefit from targeted anti-cytokine therapies, while those with a metabolic endotype could derive greater benefit from interventions addressing insulin resistance or adipokine dysregulation. By aligning treatment with the underlying biological features of each individual’s OA, this approach has the potential to improve therapeutic response rates and reduce the risk of adverse events associated with unnecessary interventions. In conclusion, while the potential for targeted therapies that address inflammation and metabolism in OA is significant, overcoming the barriers to clinical implementation will require concerted efforts from researchers, clinicians, and policymakers. Continued investigation into the dynamic interactions between these two domains is essential for advancing treatment modalities, ultimately improving patient outcomes and quality of life for those affected by osteoarthritis.
